# Prevalence and predictors of diabetes distress among adults with type 2 diabetes mellitus: a facility-based cross-sectional study of Bangladesh

**DOI:** 10.1186/s12902-022-00938-3

**Published:** 2022-01-23

**Authors:** A. B. M. Kamrul-Hasan, Mohammad Abdul Hannan, Md Asaduzzaman, Mohammad Motiur Rahman, Muhammad Shah Alam, Mohammad Nurul Amin, Mohammed Ruhul Kabir, Palash Kumar Chanda, Nadia Jannat, Md Zahurul Haque, Sanjoy Ranjon Banik, Mohammad Jahid Hasan, Shahjada Selim

**Affiliations:** 1 Department of Endocrinology, Mymensingh Medical College, Mymensingh, 2207 Bangladesh; 2 Department of Endocrinology, North East Medical College, Sylhet, Bangladesh; 3 Department of Endocrinology, Shaheed Sheikh Abu Naser Specialized Hospital, Khulna, Bangladesh; 4 Department of Medicine, Rajshahi Medical College Hospital, Rajshahi, Bangladesh; 5 Department of Medicine, Army Medical College, Cumilla, Bangladesh; 6 Department of Endocrinology, Mugda Medical College, Dhaka, Bangladesh; 7 Department of Medicine, Sylhet MAG Osmani Medical College, Sylhet, Bangladesh; 8 Department of Endocrinology, Mymensingh Medical College Hospital, Mymensingh, Bangladesh; 9 Department of Endocrinology, Aalok Healthcare & Hospital Ltd., Dhaka, Bangladesh; 10 Department of Chronic Diseases, Government Homeopathic Medical College, Dhaka, Bangladesh; 11 Pi research consultancy center, Dhaka, Bangladesh; 12 Department of Endocrinology, Bangabandhu Sheikh Mujib Medical University, Dhaka, Bangladesh

**Keywords:** T2DM, Diabetes distress, Depression, DDS-17, PHQ-9

## Abstract

**Introduction:**

Diabetes distress (DD) is common and has considerable impacts on diabetes management. Unfortunately, DD is less discussed and frequently underestimated. This study evaluated the prevalence and predictors of DD in adults with type 2 diabetes mellitus (T2DM).

**Methods:**

A cross-sectional study was conducted at several specialized endocrinology outpatient clinics in Bangladesh from July 2019 to June 2020; 259 adults with T2DM participated. Participants’ DD and depression were measured using the 17-item Diabetes Distress Scale (DDS-17) and 9-item Patient Health Questionnaire (PHQ-9), respectively. DDS-17 scores ≥2 and PHQ-9 scores ≥10 were the cutoffs for DD and significant depression, respectively.

**Results:**

The mean (±SD) age of the participants was 50.36 (±12.7) years, with the majority (54.8%) being male; their median (IQR) duration of diabetes was 6 (3–11) years. Among the study participants, 52.5% had DD (29.7% moderate and 22.8% high DD). The prevalence of emotional burden, physician-related distress, regimen-related distress, and interpersonal distress was 68.7, 28.6, 66, and 37.7%, respectively. Depression was present in 40.5%; 28.6% of the participants had DD and depression. The total DDS-17 score was positively correlated with the PHQ-9 score (*r* = 0.325, *p* < 0.001). Rural residence (OR 1.94), presence of any diabetic complication (OR 3.125), insulin use (OR 2.687), and presence of major depression (OR 4.753) were positive predictors of DD. In contrast, age ≥ 40 years at diabetes diagnosis (OR 0.047) and diabetes duration of > 10 years (OR 0.240) were negative predictors of DD (*p* < 0.05 in all instances).

**Conclusions:**

The prevalence of DD in our setting is notably high; DD and depression frequently overlap. Screening for diabetes distress may be considered, especially in high-risk patients**.**

## Background

The term ‘diabetes distress’ (DD) refers to an emotional response characterized by extreme apprehension, discomfort, or dejection due to the perceived inability to cope with the challenges and demands of living with diabetes mellitus (DM) [1]. DD includes a wide range of emotions, such as negative feelings, anger, fear, guilt, frustration, and shame, that may arise from the negative emotional burden of diabetes and concerns of the patient about blood sugar control, existing comorbidity, presence of complications, the indication of complications and access to treatments [2, 3]. DD is a common psychological state found in persons with DM and their caregivers [1]. A meta-analysis found an overall prevalence of 36% for DD in patients with type 2 DM (T2DM) [2]. DD has a negative association with a healthy lifestyle, self-management, self-efficacy, self-care, and adherence to the recommended treatment regimen, leading to worsening T2DM [2, 4, 5]. It is also directly related to cardiovascular disorders and high mortality [6, 7]. Depression is also highly prevalent in T2DM and is associated with poor glycemic control, higher rates of complications, and mortality in these patients [8, 9]. Although depression and DD are distinct conditions, DD considerably overlaps with the symptoms of major depression [10]. It is hypothesized that DD is at the milder end (but specific to diabetes), and depression is at the more extreme but general end of the spectrum of mental health problems [10]. The coexistence of the two conditions is not uncommon, and the prevalence of diabetes distress is higher in samples with a higher prevalence of comorbid depressive symptoms [2]. Management of DD is nonpharmacological, emphasizing empathic and confidence-building communication by members of the diabetes care team, whereas major depression frequently warrants drug treatment [1].

In Bangladesh, 8.4 million adults lived with diabetes in 2019 and are projected to double by 2045 [11]. Although some studies have been conducted relating T2DM and depression here, data are scarce exploring the prevalence of DD and factors associated with DD [12, 13]. This multicenter, clinic-based study was conducted to fill this knowledge gap.

## Materials and methods

### Ethical considerations

The Institutional Review Board of Mymensingh Medical College, Mymensingh, Bangladesh provided ethical approval for this study. All eligible participants were informed of the study’s aims and details about the study procedures. Informed written consent was ensured before final inclusion. As stated in the Declaration of Helsinki, the principles of biomedical research were followed while conducting the study.

### Study settings and participants

This cross-sectional study was conducted at several specialized endocrinology outpatient clinics in Bangladesh from July 2019 to June 2020. A convenient sampling strategy was performed on all adult patients diagnosed with T2DM for at least one year to collect samples. Exclusion criteria were type 1 diabetes, pregnancy, lactation, acute illness, any previously diagnosed psychiatric disorder, dementia, use of psychotropic medications, use of medications that may impair memory or cognition, and experience an unpleasant event such as a relative’s death during the prior three months.

### Demographic and clinical variables

The variables of age, gender, residence, education level, monthly income, smoking status, diabetes in first-degree relatives, age at diagnosis of DM, duration of DM, type of glucose-lowering medications, presence of diabetic complications, and comorbid illness were documented by a predesigned questionnaire and were completed by patients in a quiet room.

Participants’ physical activity-related information was documented using the Global Physical Activity Questionnaire (GPAQ) version 2. It collects information on physical activity participation in three settings (or domains), e.g., activity at work, travels to and from places, recreational activities, and sedentary behavior, comprising 16 questions. The total time spent in physical activity during a typical week and the intensity of the physical activity were considered to calculate a categorical indicator. Throughout a week, including activity for work, during transport, and leisure time, adults should do at least an equivalent combination of moderate- and vigorous-intensity physical activity achieving at least 600 MET (metabolic equivalents)-minutes [14]. The Perceived Dietary Adherence Questionnaire (PDAQ) was used to measure the participants’ perceptions of their dietary adherence. The PDAQ consists of nine questions focusing on the consumption of foods during the previous seven days. The response is based on a seven-point Likert scale to answer the question. The total score quantified the extent to which individuals perceived themselves as adhering to dietary guidelines, with higher scores indicating higher perceived adherence [15].

Standing height was measured to within 1 mm without shoes using wall-mounted stadiometers. Body weight, standing height, and waist circumference were measured, and body mass index (BMI) was calculated. We used BMI categories applicable to Asian Indians to determine the obesity status; WC ≥90 cm in males and ≥ 80 cm in females was the cutoff for abdominal obesity [16]. Recent (within the preceding month) HbA1c, serum creatinine, urine routine examination (R/E), and fasting lipid profile were retrieved from participants’ medical records. An HbA1c < 7% was defined as controlled DM. The estimated glomerular filtration rate (eGFR) was calculated using the CKD-EPI formula. An eGFR < 60 ml/min/1.73 m^2^ and/or albuminuria in urine R/E was defined as chronic kidney disease. Dyslipidemia was defined according to cutoffs described in the Adult Treatment Panel (ATP) III guidelines [17].

### Assessment of diabetes distress

Diabetes distress was measured using the 17-item Diabetes Distress Scale (DDS-17) [18]. The DDS-17 assesses diabetes-related difficulties and problems during the preceding month on a Likert scale ranging from 1 (no problem) to 6 (serious problem) [18]. The DDS provides a total DD score and four subscale scores, addressing emotional burden (five items), physician-related distress (four items), regimen-related distress (five items), and diabetes-related interpersonal distress (three items). Each subscale was scored separately by dividing the sum of its item scores by the number of items. Additionally, the mean total distress score was calculated by calculating the sum of the 17 items and dividing by 17. A mean item score of < 2.0 indicates little or no distress, 2.0–2.9 indicates moderate distress, and ≥ 3.0 indicates high distress [19]. DD was considered a dichotomous variable in this study, with patients considered to have DD if DDS-17 scores were ≥ 2.

### Assessment of depression

Depression in the participants was measured by the 9-item Patient Health Questionnaire (PHQ), which refers to symptoms experienced during the last two weeks [20]. The PHQ-9 uses a four-point Likert scale ranging from 0 (not at all) to 3 (most days) for each question, the frequency with which they have experienced specific depression symptoms in the preceding two weeks, with a total score ranging from 0 to 27. PHQ-9 scores with cutoff points of 5, 10, 15, and 20 represent mild, moderate, moderately severe, and severe depression, respectively [20]. A PHQ-9 score ≥ 10 was found to have a sensitivity of 88% and a specificity of 88% for major depression [21]. In this study, depression was considered a dichotomous variable, with patients deemed significant depression if PHQ-9 scores were ≥ 10.

### Statistical analysis

We analyzed data using Statistical Product and Service Solutions version 26.0 software (IBM Corp. Released 2019. IBM SPSS Statistics for Windows, Version 26.0. Armonk, NY: IBM Corp). Categorical variables are represented as percentages, and continuous variables are presented as the mean ± standard deviation (SD) or median (interquartile range, IQR). Student’s *t* test, Chi-square test, and Mann–Whitney U test were performed to compare the variables between participants with no DD and those with DD (moderate and high). Pearson correlation test measured correlations between total DDS score and PHQ-9 score. Bivariate logistic regression analysis was performed to determine the risk factors for DS. A *P* value < 0.05 was considered statistically significant.

## Results

### Baseline characteristics of participants

Data from 259 adults with T2DM were analyzed. The mean age was 50.36 (±12.7) years, 54.8% were male, 62.5% came from urban areas, 37.8% had a higher secondary or above level of education, 8.1% were current smokers, 66.8% had a first-degree relative with DM, and 62.9% were overweight/obese. The median duration of diabetes was 6 (3–11) years, 44.4% received insulin, the mean HbA1c was 8.12% (±2.3), and 68% had uncontrolled diabetes. All but one (99.6%) had dyslipidemia, 61% had diabetic complication(s), and 39.8% had comorbid disease(s). Of them, 66.8% met WHO recommendations for physical activity; the mean total PDAQ score was 29.74 (±10.5).

### Prevalence of DD and depression

**Fig. 1:**
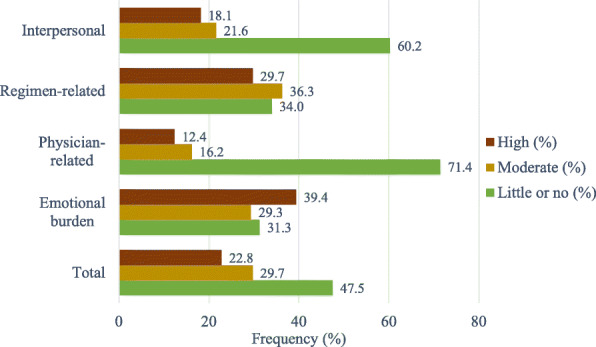
Distribution of participants according to the level of diabetes distress (*N* = 259)

**Fig. 2:**
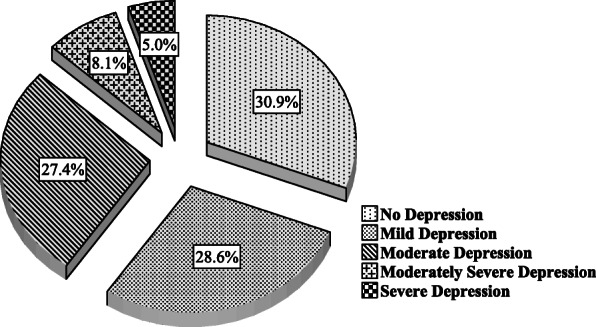
Distribution of depression among the participants (*N* = 259)

**Fig. 3:**
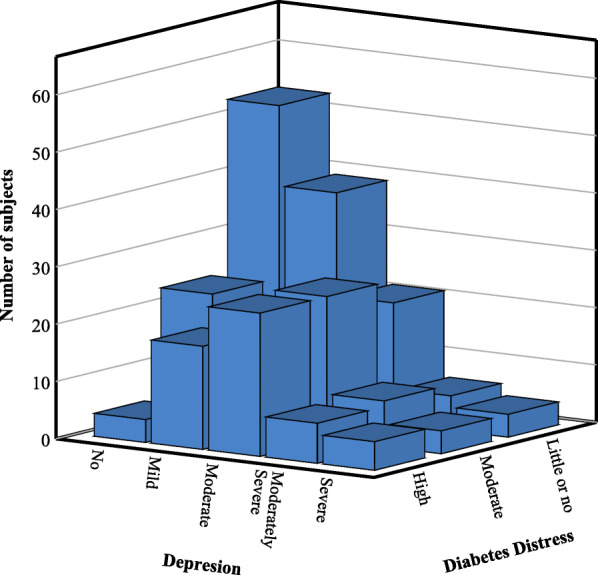
Distribution of depression within diabetes distress categories (*N* = 259)

Among the study participants, 52.5% had DD (29.7% moderate and 22.8% high DD). Emotional burden was the most crucial domain in total DD, presenting in 68.7% of the participants, followed by regimen-related distress (66%), interpersonal distress (37.7%), and physician-related distress (28.6%) (Fig. 1). Depression (PHQ-9 score ≥ 10) was present in 40.5% of the participants (moderate, moderately severe, and severe depression 27.4, 8.1, and 5%, respectively) (Fig. 2). A total of 28.6% of the participants had both DD and depression. The total DDS score had significant positive correlations (*r* = 0.325, *p* < 0.001) with the PHQ-9 score. The distribution of depression within diabetes distress categories is depicted in Fig. 3. DD was present in 70.5% of the participants with depression, whereas among those with moderate and high DD, 54.4% had depression; 62.5% of those with high DD also had depression.

### Factors associated with DD

A comparison of the variables between participants with no DD with moderate and severe DD is shown in Table 1. HbA1c, PHQ-9 score, frequencies of insulin use, participants with uncontrolled diabetes, participants with at least one diabetic complication, participants not meeting WHO recommendations for physical activity, and participants with depression were higher in the moderate and severe distress groups than in the no diabetic distress group; eGFR was lower in the moderate and severe distress groups. Participants in the two groups were similar in age, duration of DM, BMI, WC, serum creatinine, serum lipids, PQAQ scores, and the frequencies of male participants, current smoker, first-degree relative(s) with DM, overweight/obesity, abdominal obesity, and comorbidity.

**Table 1:** Sociodemographic, clinical, and biochemical characteristics of the participants according to the DD categories (*N* = 259)

Variables	Subgroups	All subjects (***N*** = 259)	Without DD (DDS < 2) (***n*** = 123)	With DD (DDS ≥ 2) (***n*** = 136)	***p*** value
mean ± SD or median (IQR) or n(%)					
Age (years)		50.36 ± 12.7	50.50 ± 11.9	50.22 ± 13.4	0.858
Age group	< 50 years	121 (46.7)	58 (47.2)	63 (46.3)	0.901
	≥50 years	138 (53.3)	65 (52.8)	73 (53.7)	
Gender	Male	142 (54.8)	72 (58.5)	70 (51.5)	0.263
	Female	117 (45.2)	51 (41.5)	66 (48.5)	
Residence	Urban	162 (62.5)	86 (69.9)	76 (55.9)	0.021
	Rural	97 (37.5)	37 (30.1)	60 (44.1)	
Monthly income category (in BDT)	< 20,000	99 (38.2)	46 (37.4)	53 (39.0)	0.482
	20,000-40,000	107 (41.3)	55 (44.7)	52 (38.2)	
	≥40,000	53 (20.5)	22 (17.9)	31 (22.8)	
Education	< Higher secondary	161 (62.2)	74 (60.2)	87 (64)	0.608
	≥ Higher secondary	98 (37.8)	49 (39.8)	49 (36)	
Current smoker	No	238 (91.9)	111 (90.2)	127 (93.4)	0.373
	Yes	21 (8.1)	12 (9.8)	9 (6.6)	
DM in First-Degree Relative	No	86 (33.2)	41 (33.3)	45 (33.1)	1.000
	Yes	173 (66.8)	82 (66.7)	91 (66.9)	
Age at diagnosis of DM	< 40 years	105 (40.5)	46 (37.4)	59 (43.4)	0.375
	≥40 years	154 (59.5)	77 (62.6)	77 (56.6)	
Duration of DM (years)		6 (3–11)	6 (2–11)	5.5 (3–10)	0.967
Duration of DM	1–5 years	129 (49.8)	61 (49.6)	68 (50)	0.780
	> 5–10 years	65 (25.1)	29 (23.6)	36 (26.5)	
	> 10 years	65 (25.1)	33 (26.8)	32 (23.5)	
BMI (kg/m^2^)		24.04 ± 3.3	24.16 ± 2.7	23.9 ± 3.7	0.560
Overweight or obese		163 (62.9)	80 (65)	83 (61)	0.522
WC (cm)		87.2 ± 9.3	87.9 ± 7.8	86.6 ± 10.5	0.271
Abdominal obesity		161 (62.2)	78 (63.4)	83 (61)	0.702
HbA1c (%)		8.12 ± 2.3	7.72 ± 2.0	8.49 ± 2.5	0.008
Control of DM	Controlled	83 (32)	48 (39)	35 (25.7)	0.024
	Uncontrolled	176 (68)	75 (61)	101 (74.3)	
Treatment of DM	Lifestyle only	5 (1.9)	1 (0.8)	4 (2.9)	< 0.001
	Lifestyle + OAD	139 (53.7)	84 (68.3)	55 (40.4)	
	Lifestyle + insulin	65 (25.1)	23 (18.7)	42 (30.9)	
	Lifestyle + OAD + insulin	50 (19.3)	15 (12.2)	35 (25.7)	
Serum creatinine (mg/dL)		0.9 (0.8–1.2)	0.9 (0.8–1.2)	0.95 (0.8–1.2)	0.643
Estimated GFR (mL/min/1.73 m^2^)		81.9 (60.1–101.4)	86.5 (60.5–102.1)	79.0 (58.6–98.7)	0.040
Total cholesterol (mg/dL)		197.4 ± 39.1	198.8 ± 36.8	196.2 ± 41.2	0.589
HDL cholesterol (mg/dL)		39.9 ± 7.5	40.4 ± 6.6	39.4 ± 8.2	0.255
LDL cholesterol (mg/dL)		127.3 ± 34.7	127.7 ± 33.7	126.9 ± 35.6	0.836
Triglyceride (mg/dL)		188.9 ± 60.9	179.9 ± 59.9	197.1 ± 60.9	0.023
Dyslipidemia		258 (99.6)	123 (100)	135 (99.3)	1.000
Any diabetic complication		158 (61)	60 (48.8)	98 (72.1)	< 0.001
Nephropathy		92 (35.5)	36 (29.3)	56 (41.2)	0.052
Retinopathy		48 (18.5)	16 (13)	32 (23.5)	0.037
Neuropathy		64 (24.7)	22 (17.9)	42 (30.9)	0.021
IHD		44 (17.0)	21 (17.1)	23 (16.9)	1.000
Stroke		5 (1.9)	3 (2.4)	2 (1.5)	0.671
Foot ulcer		9 (3.5)	2 (1.6)	7 (5.1)	0.177
Recurrent infections		24 (9.3)	5 (4.1)	19 (14)	0.009
Any comorbidity		103 (39.8)	49 (39.8)	54 (39.7)	1.000
HTN		91 (35.1)	46 (37.4)	45 (33.1)	0.515
Meets WHO recommendations on physical activity	Yes	173 (66.8)	90 (73.2)	83 (61)	0.047
	No	86 (33.2)	33 (26.8)	53 (39)	
PDAQ Total Score		29.74 ± 10.5	30.68 ± 10.6	28.89 ± 10.3	0.169
PHQ-9 Score		8.54 ± 5.8	6.98 ± 5.2	9.96 ± 5.4	< 0.001
Major depression	Absent	154 (59.5)	92 (74.8)	62 (45.6)	< 0.001
	Present	105 (40.5)	31 (25.2)	74 (54.4)	

*p* values by Student’s *t* test, Chi-square test, or Mann–Whitney U test as applicable

*BDT* Bangladeshi Taka, *BMI* Body mass index, *WC* Waist circumference, *HDL* High-density lipoprotein, *LDL* Low-density lipoprotein, *IHD* Ischemic heart disease, *HTN* Hypertension

In bivariate logistic regression analysis, rural residence (OR 1.940), presence of any diabetic complication (OR 3.125), insulin use (OR 2.687), and presence of major depression (OR 4.753) were associated with greater odds of DD, whereas higher age (≥40 years) at diabetes diagnosis (OR 0.403) and longer duration (> 10 years) of diabetes (OR 0.240) were associated with lower odds of DD (Table 2).

**Table 2:** Risk factors of moderate and high diabetes distress (DDS ≥2) among the participants (*N* = 259)

Variables	Subgroups	Odds Ratio (95% Confidence Interval)	***p*** value
Age Group	< 50 years	Referent	
	≥50 years	1.808 (0.735–4.448)	0.197
Gender	Male	Referent	
	Female	0.926 (0.462–1.860)	0.830
Residence	Urban	Referent	
	Rural	1.940 (1.003–3.752)	0.049
Income Category (in BDT)	< 20,000	Referent	
	20,000 to < 40,000	0.965 (0.483–1.927)	0.920
	40,000 and Above	2.199 (0.846–5.718)	0.106
Education Category	Up to Secondary	Referent	
	≥Higher Secondary	1.042 (0.484–2.242)	0.917
Current Smoker	No	Referent	
	Yes	0.318 (0.100–1.010)	0.052
T2DM in the First-Degree Relative	No	Referent	
	Yes	1.145 (0.595–2.204)	0.686
Age at DM Diagnosis	< 40 years	Referent	
	40 years or above	0.403 (0.164–0.988)	0.047
T2DM Duration	1–5 years	Referent	
	> 5–10 years	0.701 (0.325–1.512)	0.365
	> 10 years	0.240 (0.100–0.577)	0.001
Overweight or Obese	No	Referent	
	Yes	0.561 (0.293–1.074)	0.081
Abdominal Obesity	No	Referent	
	Yes	1.078 (0.552–2.108)	0.902
Glycemic Status	HbA1c < 7%	Referent	
	HbA1c ≥7%	1.071 (0.546–2.101)	0.825
Any Diabetic Complication	Absent	Referent	
	Present	3.125 (1.546–6.320)	0.002
Any Comorbidity	Absent	Referent	
	Present	0.602 (0.301–1.204)	0.151
PDAQ total score		0.993 (0.964–1.022)	0.619
Meets WHO recommendations on physical activity	Yes	Referent	
	No	1.781 (0.921–3.444)	0.086
Injects Insulin	No	Referent	
	Yes	2.687 (1.414–5.105)	0.003
Major Depression	Absent	Referent	
	Present	4.753 (2.466–9.162)	< 0001

## Discussion

In our sample of patients with T2DM presenting to endocrine clinics of several hospitals in Bangladesh, we found that alomost half of the patients suffered from diabetes distress, depression and a combination of DD and depression.

The variable prevalence of DD and depression in T2DM has been reported from different countries, and the prevalence greatly varies with demographics, geographical region, and cultural backgrounds. Even the cutoffs for defining DD and depression also influence their prevalence [2]. In Bangladesh, a single-center study at a tertiary hospital observed a 48.5% prevalence of DD using the same DDS-17 scale and cutoffs similar to this study to define DD [22]. The prevalence of DD was 49.2% in Malaysia, 29.4% in Vietnam, 51.3% in the USA, and 39% in Canada using the same tool and cutoffs [7, 23–25]. Forty-two percent of the population was depressed in Malaysia using the cutoff PHQ-9 score ≥ 5 [23], and 15.3% of the study subjects had major depression (PHQ ≥10) using the PHQ-8 questionnaire [7]. Emotional burden (68.7%) and regimen-related distress (66%) were more important domains than interpersonal distress (37.7%) and physician-related distress (28.6%) in measuring DD. Similar observations were found in another context [9, 22, 24].

One-fourth of the participants had DD and major depression. Major depression was present in 70.5% of patients with DD. In contrast, 54.4% had major depression among those with DD. Major depression was associated with a greater risk of DD. Tenets of DD overlap with the symptoms of major depression; depression amplifies the psychological impact of diabetes diagnosis, resulting in increased DD [26]. Various combinations of DD and depression were present in multiple studies in different locations [7, 23]. Nanayakkara et al. and Chew et al. identified depression as a risk factor for diabetes distress, which corrobates with our studies [9, 23]. This phenomenon was also supported by a meta-analysis [2]. Both DD and depression are driven by shared underlying biological and behavioral mechanisms, such as hypothalamic–pituitary–adrenal axis activation, inflammation, sleep disturbance, inactive lifestyle, poor dietary habits, and environmental and cultural risk factors [27]. Concurrent depression and DD may aggravate functional impairment, self-management problems, poorer glycemic control, increased risk of diabetes complications, and poorer quality of life [26].

In this study, age difference was not a predictor of DD. Several studies showed that patients in the higher age group had higher DD risks, but few studies also found no significant difference between different age groups [3, 28]. Younger age was associated with a higher DDS score stated by Chew et al. [23] and could be explained by the stressors of family responsibilities, work, and financial challenges. Factors associated with DD in elderly individuals could be difficulties in self-care, comorbidities, disability and lack of socal support. Therefore, the relationship of DD with age is not straightforward and interacts with other risk factors for DD.

Similar to Huynh et al., we observed no influence of gender on the risk of DD [24]. However, few studies have reported that women are at higher risk of DD [2, 3, 9]. Women tend to have greater stress reactivity to various situations, such as stressful life and work events [25]. In the current study, residence in rural areas appeared to be a risk factor for DD. Todalabagi et al.’s observations in the Indian population support this finding [29]. Tertiary care services are only available in larger cities of Bangladesh; residing in remote rural areas may result in the feeling of deprivation of adequate support from healthcare systems, which may potentiate distress. Another factor that potentiates DD was the duration of disease, and participants with DM for > 10 years had a lower risk of DD than those with DM for 1–5 years [3, 24]. As the disease progresses, there is improvement in adjusting to the disease, learning disease management skills, and increasing patients’ awareness of the disease.

Although a negative relation was found between insulin use and DD [3], a number of studies supported that insulin use poses a higher risk of DD [9, 22], and our study also explored a similar phenomenon.

Most of our participants with and without DD had uncontrolled diabetes, which may be responsible for the failure to establish uncontrolled diabetes as a risk factor for DD. DD leads to poorer self-management and medication adherence, resulting in a higher HbA1c. On the other hand, failure to achieve glycemic control may result in feelings of ‘diabetes burnout’ and increased diabetes distress. Distress is greatly enhanced with an increasing number of complications [3, 9, 22]. The presence of complications leads to many concerns, including the cost of management, the impact on daily lifestyle, employment, relationships, etc., for patients with DM; all these may adversely affect psychological wellbeing and be associated with higher distress [6]. We did not measure the type and number of comorbidities and could not infer whether a higher number of comorbidities and specific comorbid conditions were associated with DD in our participants.

Similar to Azadbakht et al., our study did not find any influence of overweight/obesity on the risk of DD. Obesity was associated with a higher risk of DD, according to Islam et al. [22]. Obesity stigma is related to psychological distress; obese people may have negative body images and need extra care in dieting, exercise, and weight-loss medications beyond their diabetes management; all these factors may accelerate distress in them.

### Limitations of the study

The study’s cross-sectional design limits its ability to judge the causality of the relationships. Some critical variables correlated with DD were not measured, e.g., employment, marital status, family and social support, insurance status, number of prescribed drugs, attended healthcare facilities, etc. The sample size was also small. The study was conducted in specialized endocrinology centers, which usually deal with more complex referred patients; therefore, the study result may not generalize to all patients with T2DM. Although the DDS and PHQ-9 are widely used tools for measuring DD and depression, respectively, they are not validated in our setting, and only one scale was used to assess the patients. Nevertheless, this is the first multicenter study in Bangladesh exploring the prevalence and associated factors of diabetes distress in the background of alarmingly increasing diabetes prevalence in the country. A wide-scale longitudinal study in this field can better explain the existing relationships between DD and other variables.

## Conclusion

Our study shows that diabetes distress is highly prevalent in our patients with T2DM. Lower age at diabetes diagnosis, rural residence, shorter duration of diabetes, presence of any diabetic complication, insulin use, and presence of major were the risk factors for DD observed in this study. The study results endorsed the need for clinical attention to DD, especially in countries with a high prevalence of T2DM, such as Bangladesh, for comprehensive and effective diabetes care.

## Data Availability

The data used to support this study are available from the corresponding author upon request (rangassmc@gmail.com).
